# Do We Swallow the Waste From Our Brain?

**DOI:** 10.3389/fnins.2021.763780

**Published:** 2021-11-23

**Authors:** Joshua Leaston, Praveen Kulkarni, Codi Gharagouzloo, Ju Qiao, Nicole Bens, Craig F. Ferris

**Affiliations:** ^1^Imaginostics, Inc., Cambridge, MA, United States; ^2^Center for Translational Neuroimaging, Northeastern University, Boston, MA, United States

**Keywords:** ferumoxytol, nasal pharynx, subarachnoid space, brain clearance, perivascular spacecan

## Abstract

Ferumoxytol, an iron oxide nanoparticle, was infused into the lateral cerebroventricle of awake rats to follow its movement and clearance from the brain using magnetic resonance imaging. Within minutes the contrast agent could be observed accumulating in the subarachnoid space, nasal cavity, nasal pharynx, and soft palate at the back of the throat. In a subsequent study fluorescent quantum dots were infused into the brain of rats and within 15 min could be observed in the esophagus using microscopy. These imaging studies clearly show that these large nanoparticle tracers (∼20 nm in diameter) leave the brain through the nasal cavity and end up in the gut. There are numerous studies going back decades reporting the clearance of tracers put directly into the brain. While these studies show the slow accumulation of trace in the blood and lymphatics, they report only accounting for less than 50% of what was originally put in the brain.

## Introduction

In a recent study we infused a small molecular weight magnetic resonance imaging (MRI) contrast agent (CA) gadobenate dimeglumine (1.06 kDa) into the lateral cerebral ventricle (LCV) of awake rats during the scanning session and mapped the site-specific movement of trace from the perivascular system into the surrounding brain parenchyma (Cai et al., 2020). This study provided clear evidence of circadian regulation of perivascular clearance that was greatest during the light phase (rat normal rest/sleep phase) but independent of sleep (Ferris, 2021). The present study was designed to repeat this study but with a much larger MRI CA ferumoxytol (731 kD) with the intention of mapping the site-specific changes in the perivascular volume over the circadian light–dark cycle. Ferumoxytol is used with quantitative ultra-short time-to-echo, contrast-enhanced (QUTE-CE) MRI, a technology we developed to measure microvascular density and function in awake rats using optimized 3D ultra-short time-to-echo (UTE) MRI and intravascular CA (Gharagouzloo et al., 2015, 2017). We assumed the ferumoxytol, a superparamagnetic iron oxide nanoparticle (SPION) with a dextran coating and 23-nm hydrodynamic diameter, would be confined to the cerebrospinal fluid (CSF) and provide contrast as it traversed the cerebroventricular system, meninges, and perivascular spaces of the brain (Abbott et al., 2018). Indeed, ferumoxytol is an excellent blood pool CA because it does not cross the blood–brain barrier. To our surprise, the ferumoxytol appeared outside the brain within minutes after administration accumulating on the soft palate at the back of the throat.

## Materials and Methods

### Animals

Subjects were all adult Sprague Dawley rats (*n* = 13), approximately 100 days of age and purchased from Charles River Laboratories (Wilmington, MA, United States). Animals were housed in Plexiglas cages and maintained in ambient temperature (22–24°C) on a 12:12 light–dark cycle (lights on at 07:00 a.m.). Food and water were provided *ad libitum*. All methods and procedures described were approved by the Northeastern University Institutional Animal Care and Use Committee (IACUC). The Northeastern facility is Association for Assessment and Accreditation of Laboratory Animal Care (AAALAC) accredited with Office of Laboratory Animal Welfare (OLAW) Assurance and is registered with the US Department of Agriculture (USDA). All housing, care, and use followed the Guide for the Care and Use of Laboratory Animals (8th Addition) and the Animal Welfare Act. The protocols used in this study adhere to the Animal Research: Reporting of *In Vivo* Experiments (ARRIVE) guidelines for reporting *in vivo* experiments in animal research (Kilkenny et al., 2010).

### Acclimation for Awake Imaging

All studies were done in awake rats to avoid the confound of anesthesia that impairs perivascular clearance (Gakuba et al., 2018). Rats underwent 5 days of consecutive acclimation. Rats were lightly anesthetized with isoflurane and placed into a copy of the restraining system used during awake imaging. When fully conscious, the animals were placed into a dark mock scanner tube with a sound recording of a standard MRI pulse sequence playing in the background. This acclimation procedure has been shown to significantly reduce plasma cortisol, respiration, heart rate, and motor movements when compared with the first day of acclimation. The reduction in autonomic and somatic response measures of arousal and stress improves the signal resolution and MR image quality (Ferris et al., 2011).

### Surgical Procedure

Just prior to imaging, rats were anesthetized with 2–3% isoflurane and received a subcutaneous (SC) injection of the analgesic Metacam (meloxicam, 5 mg/ml solution) at a dose of 1 mg/kg. The scalp was incised, and a burr hole was made in the skull for implantation of sterile PE10 tubing (Braintree Scientific) aimed at the right lateral cerebroventricle using the stereotaxic coordinates 1.0 mm posterior to the bregma, 2.0 mm lateral to the midline, and 4.0 mm in depth from the dura. The tubing, ∼60 cm in length and prefilled with ferumoxytol (Feraheme™, 731-kD) CA, was fixed in place with cyanoacrylic cement and connected to a 0.3-ml syringe needle filled with the CA that could be positioned just outside the bore of the magnet to give 10 μl of ferumoxytol (6 μg/μl Fe). This injection method has been used in previous studies to deliver drugs directly to the brain during awake imaging (Febo et al., 2004; Ferris et al., 2015).

### Imaging Acquisition

Rats were imaged within the first 4 h of the onset of the light--dark cycle. MRI was performed on a Bruker BioSpec 7-T/20-cm USR MRI spectrometer controlled by ParaVision 6.0 software. Radio frequency signals were sent and received with a custom quadrature volume coil built into the animal restrainer (Ekam Imaging Boston, MA, United States). Immediately after surgery, rats were quickly placed into the head coil and restraining system, a procedure that takes less than a minute.^1^ The design of the restraining system includes a padded head support obviating the need for ear bars helping to reduce animal discomfort while minimizing motion artifact. Scans in both axial and sagittal views were collected before and after CA administration. QUTE-CE MRI technology was used to acquire data on CSF clearance in six subjects. A radial 3D UTE sequence implementation with acquisition parameters of TE/TR/FA = 10 μs/4 ms/15°, BW = 200 kHz, 3 cm × 3 cm × 3 cm FOV, 180 × 180 × 180 matrix size, and 101,381 radial projections, was taken to produce 167 μm^3^ isotropic resolution images, with a total scan time of 7 min per image. A total volume of 10 μl of CA was injected into the lateral ventricle at a rate of 1.6 μl/min using a syringe pump. This rate of injection is reported to keep intracranial pressure within a normal range (Yang et al., 2013).

**FIGURE 1:**
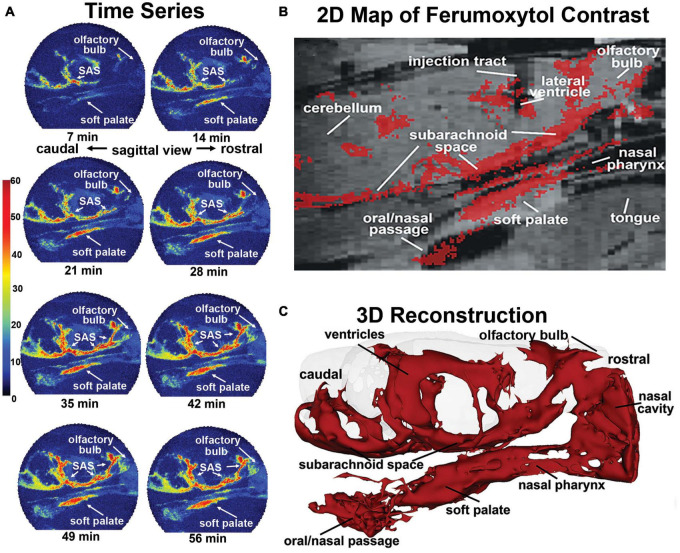
Sagittal reconstructions of ferumoxytol movement and accumulation. The time series **(A)** are images from a single rat collected at 7-min intervals over 70 min showing accumulation of CA along the soft palate over time. A summary image **(B)** generated as an average of CA signal from six rats is mapped onto the T2 weighted anatomy image of the brain. **(C)** The 3D reconstruction of CA within and outside the brain was generated from a single rat, post-mortem, at the end of the imaging study to eliminate the artifact of blood vessels.

Contrast-to-noise ratio (CNR) maps as defined by signal-to-noise ratio (SNR) post-contrast subtracted by SNR pre-contrast were co-registered to normalized space and generated with MATLAB as demonstrated in Figure 1. A region of interest within the field of view in air outside the head was used to obtain the standard deviation of the noise in calculating SNR. All other segmentations were rendered in 3D-Slicer (Fedorov et al., 2012). One subject underwent an additional 10 scans post-mortem, having died at the end of the imaging session while still in the scanner. These data were utilized to produce a high-resolution 3D segmentation irrespective of blood flow contributions and motion artifact.

### Quantum Dots

A series of studies were run on female and male rats using quantum dots [Qdot^®^ 605 ITK™ amino (PEG)] with a hydrodynamic diameter of 16–20 nm. While under light isoflurane anesthesia, three female rats were infused for 2 min with 20 μl of Qdots into the LCV. One female and one male rat were infused with 10 μl of Qdots mirroring the QUTE-CE imaging protocol. One male rat was infused with 0.9% NaCl vehicle at 10 μl/min, and one male was given a 1-μl injection of Qdots into each nostril. They were allowed to awaken and, between 15 and 40 min post infusion, euthanized; the upper esophagus was removed and bisected along the long axis on a microscope slide, and the tissue was compressed under a coverslip. Slides were imaged using a Zeiss LSM 880 confocal microscope equipped with a Plan-Apochromat × 63/1.4 Oil DIC M27 objective. The Quantum Dots were excited with a 405 laser at 10% laser power. Accompanying the fluorescent image was a brightfield image acquired to provide context and locational information within the tissue sample.

## Results

This observation was made in six rats with cannula placements in the LCV and infused with 10 μl of ferumoxytol (6 μg/μl Fe) during the MRI scanning session while fully awake. QUTE-CE images were collected continuously at 7-min blocks for a duration of 70 min. Shown in Figure 1 are sagittal sections from one rat depicting the accumulation and localization of ferumoxytol signal following intracerebroventricular (ICV) infusion. All rats tested showed a similar signal profile (see Supplementary Figure 1). The images in Figure 1A are a times series of voxel-based maximum intensity projections (MIPs) made from the sagittal view of the entire rat head taken at 7-min intervals. The color scale represents the CNR, as defined by the signal-noise-ratio post-contrast, subtracted by the signal-noise-ratio pre-contrast. Note the accumulation of ferumoxytol at the soft palate, olfactory bulb, and subarachnoid space (SAS) over time. Interestingly, trace can be observed accumulating on the soft palate as early as 7 min post ICV infusion attesting to its rapid clearance from the brain. The appearance of trace outside the brain in blood or lymph nodes following injection ICV, intrathecally, or into different brain regions, has been reported in numerous studies. However, most CSF clearance studies in animals have been performed under anesthesia with reports of trace appearing in blood and lymph nodes between 30 and 180 min post administration, e.g., dog (Di Chiro et al., 1986), rabbit (Bradbury et al., 1981), cat (Pile-Spellman et al., 1984); mouse (Aspelund et al., 2015; Ahn et al., 2019), and rat (Szentistvanyi et al., 1984).

Figure 1B shows a summary image of all the scans from all six rats using a voxel threshold CNR greater than 5. These data are mapped onto the corresponding average anatomical image for all rats. Note the injection site in the LCV. Ferumoxytol accumulates along the base of the brain in the SAS while there is no evidence of accumulation of CA on the dorsal surface of the brain as noted in previous studies (Ahn et al., 2019). The presence of CA on the rostral surface of the olfactory bulb reflects the movement from the SAS through the cribriform plates and below into the nasal epithelium and possibly the nasal cavity. The olfactory route of CSF clearance through the cribriform plate and into the nasal mucosal lymphatics to deep cervical lymph nodes is well documented across multiple mammalian species (Pile-Spellman et al., 1984; Johnston et al., 2004; Aspelund et al., 2015; Norwood et al., 2019). CA can be seen all along the boundaries of the nasal pharynx leading down to the soft palate and oral/nasal passage. Figure 1C shows an average QUTE-CE image generated from 10 scans with a transparent overlay of the brain for anatomical reference (70 min of image acquisition time). This 3D summary of ferumoxytol movement and localization following ICV infusion highlights the cerebroventricular system, the caudal/rostral extent of the SAS, movement across the olfactory bulb and into the nasal cavity, and down the nasal pharynx to soft palate and oral/nasal passage.

**FIGURE 2:**
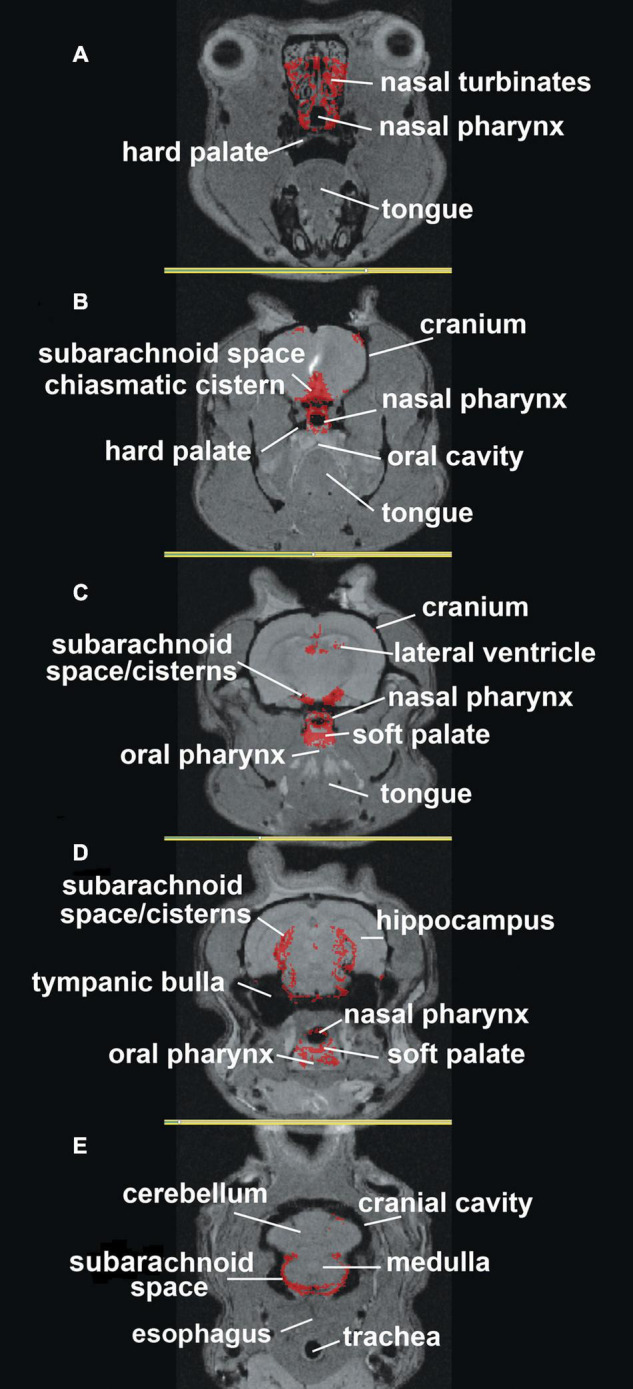
Mapping ferumoxytol signal. Shown are axial sections **(A–E)** depicting the localization of the average CA signal from six rats mapped onto T2 weighted anatomy images of the rat brain.

The localization of ferumoxytol to anatomical brain areas is better shown in Figure 2, where the accumulation of CA is mapped onto high-resolution MRI images. The most rostral section 2A shows CA around the nasal turbinates and nasal pharynx. Caudal sections 2B through 2E show CA along the basal lateral SAS and cisterns. There is CA around the nasal pharynx (2B,C) with accumulation starting on the soft palate (2C) and oral pharynx (2D). The most caudal section 2E shows CA around the SAS at the level of the cerebellum and medulla but missing from the trachea and esophagus. The field of view during the scanning session did not allow for imaging of the deep cervical lymph nodes which would have been further caudal to section 2E. This visual record of ferumoxytol accumulation over the first hour after ICV infusion was similar across all six rats tested (Supplementary Figure 2).

### Clearance Through the Nasal Cavity

All studies to date have assumed that CSF leaving through the cribriform plates is taken up by the lymphatic vessels in the mucosal lining of the nasal turbinates to eventually find its way back to the systemic venous circulation (Proulx, 2021). Could contents from the CSF appear in nasal exudate? Healthy dogs show leakage of CSF into the nasal cavity that can be followed by imaging of CA injected into the brain (Di Chiro et al., 1972). This phenomenon is thought to be an anomaly unique to dogs and was given the name rhinorrhea. However, recent studies in humans have shown swabs of nasal exudates can be used to assess the differences in iron levels following brain damage from ischemic or hemorrhagic strokes (Garcia-Cabo et al., 2020) and amyloid beta from nasal secretions of patients with Alzheimer’s dementia (Kim et al., 2019). It is not surprising that biomarkers from the brain can appear in the mucus of the nasal cavity given the success of drug delivery to the brain across the same mucus boundary (Pardeshi and Belgamwar, 2013). Drugs given intranasally gain rapid access to the brain, and what is not absorbed is simply swallowed (Pardeshi and Belgamwar, 2013). Indeed, any brain-derived or exogenously inhaled material found in the mucus of the nasal cavity would be expected to move down the nasal pharynx helped by the propulsion of microcilia to eventually meet with the oral pharynx at the back of the throat. This raises an intriguing question. Could these rats in our study be swallowing a portion of ferumoxytol cleared from their brain? Previous studies have searched systemic and lymphatic circulations to account for trace injected into the brain, which can only account for less than half of what is administered (Bradbury and Cole, 1980). Perhaps the missing trace ends up in the gastrointestinal tract.

### Finding Brain-Derived Trace in the Esophagus

**FIGURE 3:**
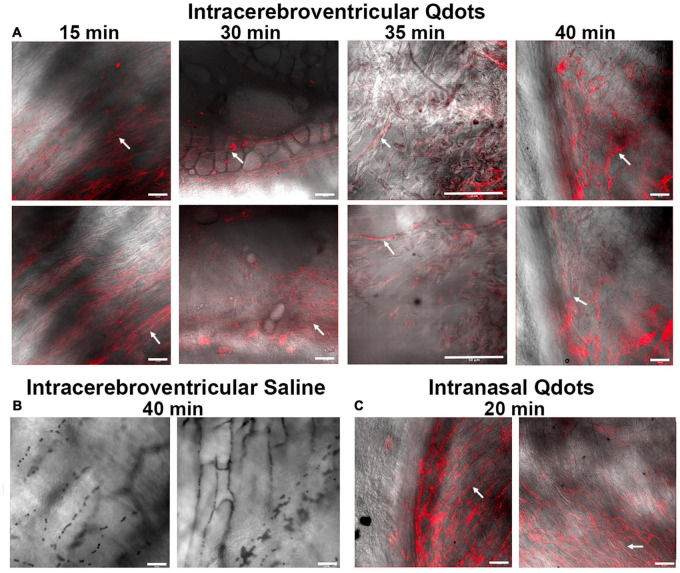
Fluorescent images of the lumen of the upper esophagus. Shown in the upper panel **(A)** are photomicrographs of the lining of the esophagus from *ex vivo* tissue taken at different times following ICV infusion of Qdots. Each time point displays two images from different areas of the esophagus. The 15-min sample was collected from a female rat infused with 10 μl of Qdots, while the 30- and 35-min samples are from females infused with 20 μl of Qdots. The 40-min data were collected from a male infused with 10 μl of Qdots. The lower left panel **(B)** shows photomicrographs of esophagus from a male rat infused with 10 μl of 0.9% NaCl (saline) taken with Zeiss LSM 880 confocal microscope held at the same fluorescent threshold as the other images. The lower right panel **(C)** shows the presence of Qdots in the esophagus of a male rat following 1-μl injections of Qdots into each nostril. White arrows point to the putative linear threads and strands of Qdot accumulating in mucus. Scale bar = 50 μm.

If the CA was being swallowed by awake rats during the scanning session, it should appear in the esophagus. To test this hypothesis, we ran a series of studies on female and male rats using quantum dots with a hydrodynamic diameter of 16–20 nm. Figure 3 shows a collage of the fluorescent images with two representative examples of each experiment at different time points. The red signal associated with the long linear threads and twisted strands (white arrows) is presumably Qdots adhering to polymers of mucin glycoproteins. Mucus glycoproteins behave as a polyanionic polyelectrolyte that can readily hydrate and bind to cationic ions (Davies et al., 2002; Thornton et al., 2008). Qdot^®^ 605 ITK™ amino with its net positive charge could be acting as a counter ion helping to bind to the mucin. Note the presence of Qdots as early as 15 min post ICV infusion (Figure 3A). The upper esophagus collected from a male rat ICV infused with 10 μl of saline (Figure 3B) showed no evidence of red fluorescent signal, while the esophagus of a male injected with 1 μl of Qdot into each nostril (Figure 3C) is replete with red fluorescent signal organized in threads and strands that match the images from ICV infused Qdot.

## Discussion

Why is the evidence so clear that ICV injected CA accumulates on the soft palate and oral/nasal pharynx with MRI, yet has never been reported before? While there are probably multiple reasons, we will focus on three.

### Attention Elsewhere

Anything escaping from the systemic circulation is taken up by the lymphatic vessels, acted upon by the reticuloendothelial system of lymph nodes and recycled to the venous circulation. The original reports of central nervous system (CNS) tracers appearing in the cervical lymph nodes across different species mirrored what was happening in the periphery and fulfilled an expectation (Proulx, 2021). One would not be expected to look in the gut for CSF waste when all the attention was justifiably focused on the cervical lymph nodes. To this point, X-ray imaging of CSF outflow 30 min after cisternal infusion of CA in rabbits shows a signal in lymph nodes in addition to the soft palate, but the latter was not discussed (Liu et al., 2012). ICV injection of Combidex, a dextran coated SPION MRI CA, in rats shows a signal in lymph nodes and an ostensible amount of signal around the soft palate and oral pharynx (Muldoon et al., 2004). Images from this study are shown in Supplementary Figure 3 depicting the injection site of CA into brain parenchyma and ICV. Both routes of administration present with bright signal below the trachea localized to the soft palate and oral pharynx. Yet again, this signal contrast was not addressed in the study.

### Confound of Anesthesia

Nearly all studies cited in this article, and others from reviews of perivascular clearance, report the use of anesthesia. ICV infused MRI CA comes to equilibrium across the brain within 30–40 min in the awake rat (Cai et al., 2020) and is rapidly cleared from the mouse brain within 15–30 min (Ma et al., 2019), but under anesthesia these processes may take hours. How much of the clearance of CA is delayed or redirected using anesthesia (Gakuba et al., 2018; Ferris, 2021)? Here we show Qdots are rapidly moved to the nasal cavity, presumably collected in mucus, and propelled by mucocilliary action down the nasal pharynx to the back of the throat where they appear in the esophagus—as early as 15 min post ICV infusion. Concentrating the rapidly cleared ferumoxytol along the nasal pharynx and soft palate improves its visibility.

### Quantitative Ultra-Short Time-to-Echo, Contrast-Enhanced Imaging

Quantitative ultra-short time-to-echo, contrast-enhanced is a recently patented imaging technology developed for quantitative vascular imaging optimized with 3D UTE pulse sequences with intravascular ferumoxytol to produce positive contrast angiographic images unparalleled to time-of-flight imaging or gadolinium-based first-pass imaging (Gharagouzloo et al., 2015; Knobloch et al., 2019). We recently published a study mapping the absolute physiological cerebral blood volume (CBV) of the awake rat brain, including measurements of microvascular density, vascular functional reserve, and CBV modulation with anesthesia (Gharagouzloo et al., 2017). This more sensitive MRI technology combined with ICV infusion and rapid accumulation of ferumoxytol in the back of the throat under awake conditions would be the most probable explanation for the observations made in this study.

### Point of Concern

Does the small opening in the cranium and the administration of CA into the brain artificially affect clearance? While the cranium is resealed and the infusion volume and rate adjusted so there is no increase in intracranial pressure, there is an inherent flaw in this experimental paradigm acknowledged by practitioners in the field as addressed in multiple publications. The past literature cited above looking at brain clearance to blood and lymphatics accepts the measurements knowing this limitation. The identification of this third route of clearance from brain to gut will require a thorough evaluation of how much and how quickly CA is cleared from the brain and the effects of changes in pressure, temperature, and volume.

## Summary

As noted above, efforts to recover tracer injected into the brain could only account for 50% or less using measurements taken from blood and lymphatics (Bradbury and Cole, 1980). Our finding that rats swallow trace infused into their brain CSF could explain the missing 50%. Moreover, the rapidity in which a large nanoparticle (∼20 nm in diameter) can move from the ventricular system of the brain to the esophagus is stunning. Future studies are needed to quantify this phenomenon, i.e., how much is moved and how quickly. However, and more importantly, do humans swallow the waste from their brain CSF? The examples provided above using nasal swabs to presumably sample biomarkers coming from the brain are suggestive but not convincing (Kim et al., 2019; Garcia-Cabo et al., 2020). Perivascular clearance in humans is greatest during the dark phase of the circadian light–dark cycle during rest and sleep. Mucus accumulates along the nasopharynx during sleep. The morning accumulation of mucus at the back of the throat may offer a rich source of brain biomarkers.

## Data Availability Statement

The original contributions presented in the study are included in the article/Supplementary Material, further inquiries can be directed to the corresponding author.

## Ethics Statement

The animal study was reviewed and approved by the Northeastern University IACUC.

## Author Contributions

CF, PK, CG, and JL: concept, drafting, and interpretation. JL, PK, JQ, CG, and NB: execution and analysis. All authors have contributed substantially to the manuscript and read and agreed to the published version of the manuscript.

## Conflict of Interest

CF has a financial interest in Animal Imaging Research, the company that makes the radiofrequency electronics and holders for awake animal imaging. CG has financial interest in Imaginostics, a company commercializing neuroimaging biomarkers of the vascular and perivascular spaces. JL was employed by company Imaginostics, Inc. The remaining authors declare that the research was conducted in the absence of any commercial or financial relationships that could be construed as a potential conflict of interest.

## Publisher’s Note

All claims expressed in this article are solely those of the authors and do not necessarily represent those of their affiliated organizations, or those of the publisher, the editors and the reviewers. Any product that may be evaluated in this article, or claim that may be made by its manufacturer, is not guaranteed or endorsed by the publisher.

## Footnotes

1
https://www.youtube.com/watch?v=JQX1wgOV3K4
