# Supplementary figures and images for: Do We Swallow the Waste From Our Brain?
Source: Front Neurosci. 2021 Nov 23;15:763780. doi: 10.3389/fnins.2021.763780 (PMC8649892; doi:10.3389/fnins.2021.763780)

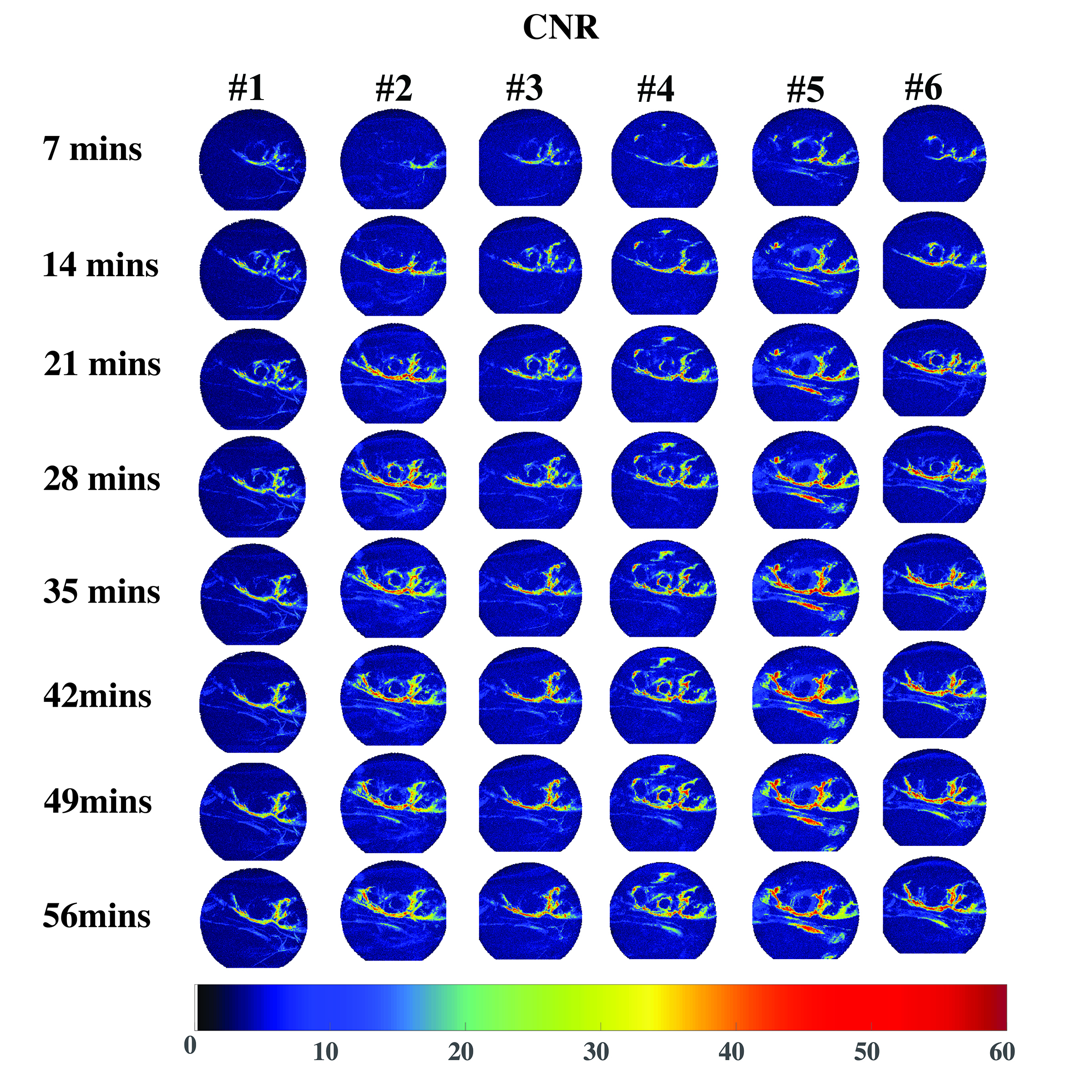

Supplement: Supplementary file 1 [file Image_1.JPEG]

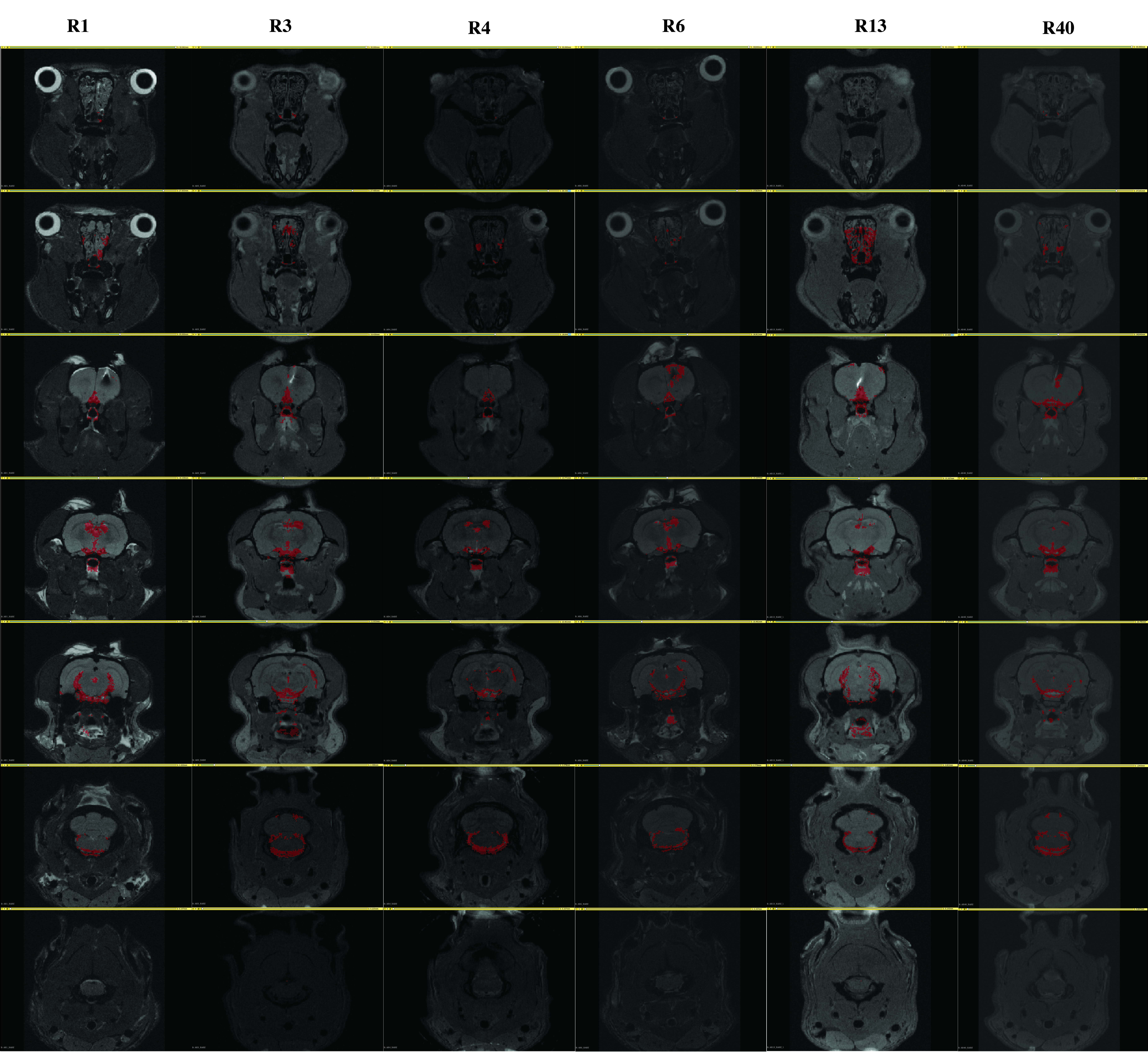

Supplement: Supplementary file 2 [file Image_2.JPEG]

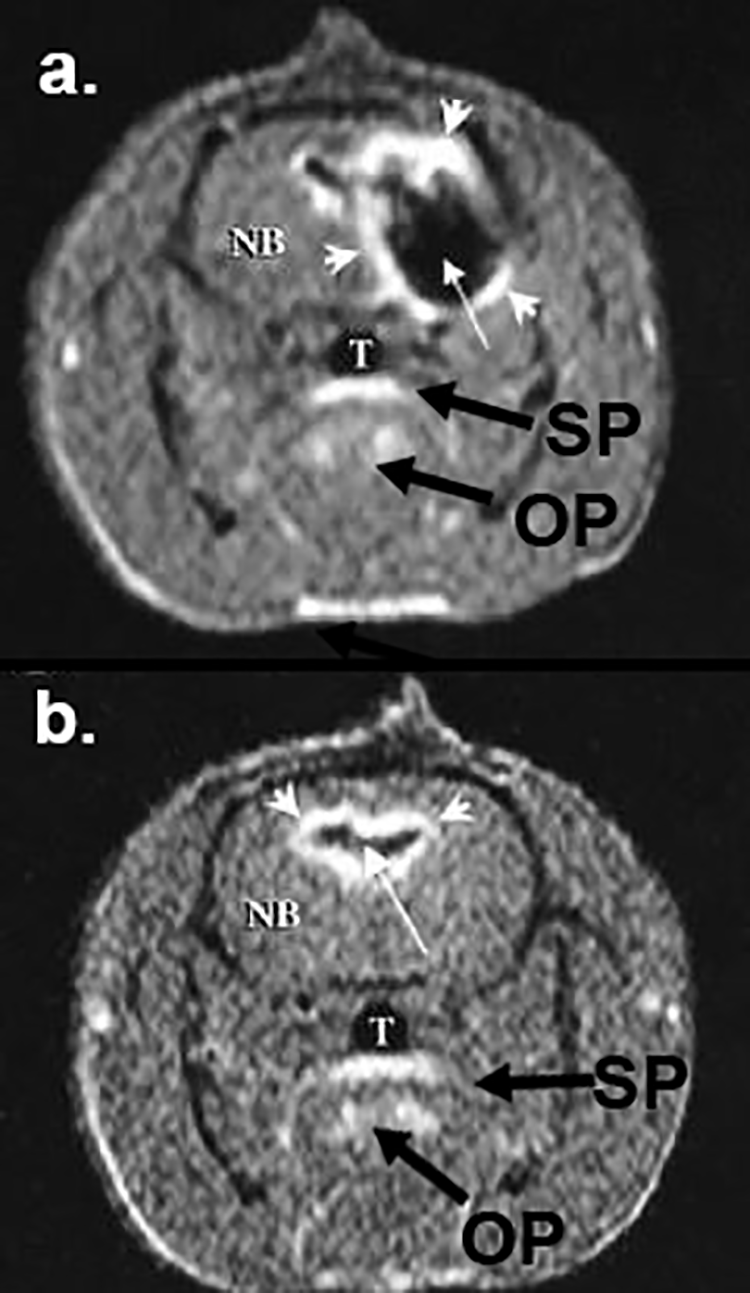

Supplement: Supplementary file 3 [file Image_3.TIF]
